# The Experience of Adult-Onset Hearing Loss and Adaptation to a Cochlear Implant

**DOI:** 10.1177/15459683251372922

**Published:** 2025-08-27

**Authors:** Bruce H. Dobkin

**Affiliations:** 1 Department of Neurology, Neurologic Rehabilitation Program, Geffen School of Medicine, University of California, Los Angeles, CA, USA

**Keywords:** auditory neuroplasticity, auditory rehabilitation, cochlear implant, hearing aid, sensorineural hearing loss

## Abstract

**Background:**

Spoken language and environmental sounds hold rich and nuanced meaning for the listener, but depend on accurate hearing of the soundscape, including the timing, volume, and contrasts of its component pitches. Sensorineural hearing loss with aging degrades these properties, leading to progressive disability.

**Objectives:**

This case study and review describe my experience and behavioral accommodations to progressive bilateral hearing loss, limited compensation with hearing aids, and the stuttering evolution of gains after a unilateral cochlear implant (CI).

**Results:**

Despite increasingly powerful hearing aids over 25 years, spoken phonemes and words became increasingly muffled, misheard, and often dissipated into ambient background noise. The cognitive effort to extract meaning and mask my disability grew exhausting. I gradually eliminated many of my usual family, medical career, and social roles. To try to recover some communication-dependent activities, I sought a bionic solution. A right-sided CI initially carried an ambiguous, fizzling code and unrecognizable synthetic voices. With 8 months of auditory rehabilitation, I better deciphered conversational speech and ambient sounds. By audiological testing, I improved from 10% hearing accuracy of single words to 65%, typical of post lingual adult users. Better hearing in ambient noise and for what had been excessively rapid speech evolved out to 18 months, allowing me to re-engage in many of my daily roles.

**Conclusions:**

Hearing loss beyond the compensation of aids deeply challenges quality of life. Auditory rehabilitation after cochlear implantation engages neuroplasticity to re-establish functional communication.

## Introduction

Sensorineural hearing loss from damage to inner and outer cochlear hair cells is the most common sensory impairment among adults. Such damage degrades the ability of auditory neurons to adapt to constant and changing stimuli, such as mean sound levels, sound contrasts, and the multiple frequencies that characterize the range of soundscapes.^1^ Quality of life suffers. The prevalence sharply increases after age 40, reaching 35% at age 70 for mild, 16% for moderate, and 3% for severe loss, then doubles for moderate-to-severe loss by age 80.^2^

I report personal and the research observations by others on how hearing and daily behavior are affected by progression toward deafness and the limits of compensation with hearing aids or a cochlear implant, as well as the remarkable neural and behavioral adaptations that are engaged by a unilateral cochlear neuroprosthesis.

## The Experience of Progressive Hearing Loss

Only about 20% of Americans whose social and work communication could benefit from the pitch amplification of hearing aids wears them.^3^ Recent public health studies also suggest that aids can reduce cognitive decline in moderately hearing-impaired persons,^3^ although they may not prevent functional decline in daily activites.^4^

I embraced hearing aids at age 45. My audition continued to wane on pure tone audiometry testing by about 2 to 3 decibels (dB) per year for several decades, similar to others with age-related and noise exposure-induced loss. My experience was also in parallel to the World Health Organization’s progressive stages of hearing loss (Table 1).^5^ My decline had been an insidious distortion of speech clarity accompanied by noticeable decrements in everyday communication every 5 years or so. Despite adding increasingly powerful hearing aids, spoken phonemes and words became increasingly muffled, misheard, and often lost in the space between the speaker and me, or simply dissipated into ambient background noise. The colorations of speech faded, like the pigments of flowers in twilight.

**Table 1.** Average Hearing Thresholds for Spoken Speech Frequencies of 500, 1000, 2000, and 4000 Hz.

Decibels required	WHO grade of hearing loss	Hearing experience
<20	Normal	No problem
20 to <35	Mild	Some difficulty with conversation in background noise
35 to <50	Moderate	Difficulty with conversational speech, worse in noise
50 to <65	Moderately severe	Hear raised voices in quiet
65 to <80	Severe	Does not hear most conversational speech and still difficult despite raised voices; poor in noise.
80 to <95	Profound	Extreme difficulty with raised voices and no discrimination in noise.
95 and greater	Complete loss	Hears no speech or environmental sound.

*Note*. Adapted from The World Health Organization and Lin^6^.

Non-native accents, whispers, high-pitched voices, and speakers who did not clearly enunciate or project each word at a slower than usual pace all came to befuddle my comprehension and patience. Learning and memory suffered under aural ambiguity. Music increasingly sounded off key; I barely recognized the instrumentals and songs of artists I once knew well. Many interacting structural and physiological changes, both adaptive and maladaptive over the time of inner and outer hair cell loss, contribute to the complex alterations of hearing perception that go far beyond a simple need for volume amplification.^6-10^

Like others before me, I tried to compensate as I declined. I moved within a few feet of speakers, asked them to face me and slow down their seemingly too rapid word pace, speak a bit louder or repeat a few words, remove a hand from near their mouth, and not whisper an aside. I often suggested we move to a nearby quieter space. As hearing declines, impaired speech reception in even modest background noise becomes a common symptom.^11^ Their response was immediate and kind, but most fell back to their normal speech pattern within a few minutes. The effort to pay close attention to word sounds and reconstruct meaning from context, especially in background noise, increasingly challenged my ability to persist in the deep concentration now necessary to get the gist of conversations and lectures. Degraded audition rattled my capacity to think about content as others spoke and too often punched silent holes in my verbal memory.

Moderate-to-severe hearing loss infiltrates all aspects of daily life and career, well beyond a spouse telling you the TV is too loud or you do not listen to what she says. By my mid-50s, I gave up eating at restaurants due to crowd noise that drowned spoken words. Soon after, I no longer accurately heard actors during live theatre performances and only watched movies at the cinema or on television if they included closed captions. I stopped asking for assistance at bustling stores. Instead of answering phone calls, I replayed recorded messages until I garnered enough meaning. Direct Bluetooth transmission of caller voices from my iPhone into my hearing aids transiently improved my waning comprehension especially when combined with real-time closed captions, despite their frequent translational errors.

After age 65, my hearing acuity dropped precipitously. At my nadir, it took 70 to 80 dB (almost a shout) to hear pure tone frequencies at 1000 Hz or greater without aids, sounds that I had appreciated at a normal 20 dB before my early forties. Hearing aid amplification with high frequency compression could not snap sound into focus with the impact of corrective lenses that restore 20/20 vision.^7^ The spoken phrase was cracking, now threatening my career. My efforts became a bit desperate. When I failed to make sense of friends and colleagues in a group discussion, I found myself emoting like an actor in a silent movie. I mimicked the cues of other listeners or the speaker—a nod, smile, frown, or raised brow, perhaps an *aah* or *hmm*. Facial mimicking is usually a tool of empathy. I wore it as camouflage.

I stopped asking questions of lecturers, because I was uncertain whether they had already addressed the subject of my query. I was mostly reading their presentation slides, not hearing up to half their words. More than once at the end of giving a lecture, I fell into confused silence in front of a large audience, unable to make clear sense of a question even after asking the attendee to repeat it. After a conspicuous pause, I might respond, “Yes, interesting,” then blindly toss out a tidbit that was probably irrelevant to the asked question. Increasingly, I avoided engaging with colleagues at professional meetings, sometimes hiding in my hotel room instead of attending conference coffee breaks and meals with their inherent murmuring noise. So I quit attending conferences, committee meetings, and giving lectures.

After COVID-19 hit in 2020 and face masks were worn, I realized how much I had depended on subconscious lip reading,^12^ especially as a visual cue for the first syllable of words. Plus, a mask reduced the projection and clarity of vocalizations. One-on-one teaching with a trainee who would present a patient to me in a quiet clinic room now became unsafe. I retired from my academic career. Even more devastating, I could not understand the words of my 4 grandkids, all under age 7, as they patiently tried to communicate. After a year reading the science behind cochlear implants and talking to recipients, I reached for a single CI.

## Recovery of Hearing With a Cochlear Implant

Almost 9000 deaf Americans received a CI in the year before the COVID-19 pandemic. In 2022, the US Department of Health and Human Services agreed to cover the cost, approximately $75 000, for highly impaired if not completely deaf Medicare recipients like me. The number of yearly CIs, then, is likely to grow, especially if prognostication about specific hearing outcomes for individuals improves and barriers lessen.^13^ What might the new users anticipate?

In 2023, an otolaryngology physician surgically placed a 1-inch diameter CI receiver with a magnet under the scalp on the skull above my right ear lobe.^14^ The device was connected under the scalp to 22 thin wire electrodes that had been pushed through a tiny hole bored into my mastoid and threaded at increasing distances along the spiral tunnel of the cochlea. Three weeks later, an audiologist magnetically clipped a 1-inch diameter outer scalp transmitter over the skull piece receiver. This outer component was wired to the microphone and speech microprocessor that curled over the cartilage of my right ear. I heard some crackles as the north and south poles met, firing up shock and awe.

The voice of the audiologist was synthetic, flat, and robotic. No melody or human expressiveness peeked through. Sounds were also chopped up as if frying in sizzling bacon grease. My speech recognition, not unexpectedly, was hit and miss. The audiologist plugged my left ear canal. She spoke slowly from a list of 10 vegetables. I missed all but 2. *Cucumber*, for instance, sounded like *salamander*. After a few repeated trials of the same list, I cautiously hit 9 of 10. The audiologist sent me off to practice.

### CI Training

Auditory rehabilitation for hearing aids or a CI involves instruction about how to adaptively optimize one’s performance and reduce the effort of listening.^15^ Internet sites are available for training (e.g., https://angelsound.tigerspeech.com), but therapist-based instruction is usually brief and informal.^16^ Highly experiential self-training and motivation to practice become essential.

Using the manufacturer’s Cochlear, Inc. training website, my wife initiated my rehabilitation by reading aloud lists of phonemes and single-syllable words for several hours a day with my left ear canal plugged. Polysyllabic words, phrases, and short sentences followed. I intuited more at each session, but could not carry over that recognition to the next day for several weeks. So we experimented. After mishearing a word or phrase several times, my wife showed me the printed word. Then she said it again. I immediately heard the correct words. That seemed magical, but the auditory pathway had been primed by the letters, my knowledge of word sounds, and the cerebral connections between graphemes and phonemes. Contextual cues about what she said (e.g., a list of furniture; a conversation that takes place in a shoe store) also helped me make a better prediction. To detect meaning from misheard sounds, I depended, as I had with hearing aids, on my prior experiences and expectations about language and content.

In the second week, my wife read aloud short portions of newspaper and magazine stories, cooking recipes, and tidbits from the Internet. I sometimes made unimaginably wrong interpretations, so off kilter that we both laughed deliciously (e.g., a recipe to *Shirt the shadows in butter*). Short sentences contained parceled sounds, like the clicking dots and dashes of rapid Morse code transmission. Phonemes did not flow into each other. Too often, bereft of contextual or visual cues, I was trying to intuit meaning in scattered word-sound entrails. Environmental sounds were almost entirely uninterpretable—lots of ka-chings, shaking castanets, and flurries of hail rapidly tapping against a metal roof. Tintinnabulation surrounded me outdoors.

Oddly, I heard at least 3 different estranged voices when anyone spoke. The left hearing aid provided a lower pitched, more melodic voice. The CI presented a higher frequency, pixilated, and hissy voice with hollow echoes. The third voice was an unmerged mix of the 2. I would get caught up in perceptual dissonance, turning my attention back and forth to the sound of the voice managed by the hearing aid or by the CI or heard the separate but simultaneous inputs. The streams gelled after 5 months of practice.

By the third week of bionic practice, the modest training resources I had accessed became less challenging. I upped the rehabilitation ante by simultaneously reading and listening to *Fahrenheit 451* piped via Bluetooth from my iPad into both of my hearing devices. With this multimodal sensory input, I listened at 60% of the usual audiobook speed to start Ray Bradberry’s masterpiece, a story I had read several times in the past. By the end, I reached 80% speed and referred to the written word less often.

After a month of training, audiologic tests revealed that I now recognized 48% of clearly enunciated single words directed into the CI in a sound-proof room, compared to barely 10% in my right ear before the CI. Much better discrimination, but still not enough for functional communication under less than ideal circumstances. The audiologist programmed higher volume for conversational frequencies and made other adjustments. Spoken words soon became more distinct, seemingly less distant. I also noticed better detection of the onset and end of individual words and sentences. But tearing a piece of paper or metal pots banging into each other startled me like a chalk screech across a blackboard, just as when my first hearing aids heralded recaptured high pitches.

I tackled a dozen more audiobooks at 75% speed to start and was able to finish later novels at 90% of the suggested pace. At 100%, phonemes and words too often bumped into each other chaotically. I had to check about 8 to 10 words per page over the first half-dozen novels, but only 4 to 5 after that. I restarted attending the 1-hour weekly neuroscience Zoom conferences presented by my neurology department. I calculated that a talking speed during these academic lectures of >160 words per minute sharply reduced my comprehension and >180 left me befuddled. As with the audiobooks, 140 words/min were in my strike zone, but most people spoke faster.

An audiology assessment at 4 months revealed that I improved to 64% single-word discrimination on the CI side. With bilateral hearing, I successfully recognized almost 90% of single words spoken directly into both the hearing aid and CI’s microphones in a quiet space. Indeed, my speech recognition with the left-sided hearing aid is still much better when combined with simultaneous CI input. Most reassuring, I had more than doubled the number of correct words and sentences with the CI plus left aid compared to using bilateral aids pre-op. For pure tones on the right, I now heard speech frequencies from 500 to 4000 Hz at the amplification of 35 to 40 dB. On its logarithmic scale, that was a 10 000-fold improvement from previously needing 80 dB. Speech picked up by the CI still had a percolating quality without the inflections, rhythms, and prosody of ordinary speech. A voice is usually as distinct and complex as the person’s signature or fingerprint, but had not yet for me, not even for the voices of my wife, daughters, and my own. I also experienced odd auditory illusions and annoying musical hallucinosis, described in a separate report.^17^

I practiced listening to all sorts of music but failed to recognize instrumentals and lyrics. I appreciated a rhythmic beat, probably either a drum or bass guitar; the rest was a chaotic blend of pitches. A Bach cello solo directed from my iPhone into both hearing devices evoked strumming a washboard. Vocalists were indistinguishable.

At 6 months, I finally recognized the distinct and familiar voices of my family, friends, and most colleagues. Some CI recipients persist in hearing only flat synthesized voices. When my wife called me, however, I always heard her off to my right and turned that way, no matter where she stood. I then did a slapstick search by as much as 360 degrees until I found her. That spatial localization of sound never improved and affects most CI users, even those with bilateral CIs.^18^

Subsequent auditory tests beyond 8 months revealed no further jump in acuity, but real-world listening comprehension did improve. The effort to mentally repair a misheard word and reconstruct the spoken sentence,^19^ which often led to missing the next phrase or sentence, diminished. Ninety percent of the learning curve for better hearing skills is achieved at 7 to 10 months, at least for what audiologists test.^20^ On average, a CI restores word discrimination at conversational loudness at 65% of normal levels during audiological testing, as I had achieved. Not all recipients obtain the same degree of benefit, partly related to cognitive factors,^21,22^ as well as adaptive and maladaptive neural plasticity^23^ and limitations in precision of CI electrode placement.^24-26^ Every recipient mislabels words, but the distortions seem specific to individuals.^27^

At 2 years post implant, I can discriminate up to 170-plus words/min in conversation, as long as others face me within 8 feet, speak at usual conversation volumes, and background noise such as chatting, music, running water, fans, street traffic, etc. is less than 50 dB. I still routinely cue speakers as I did with hearing aids to optimize my comprehension. Most environmental sounds are fully recognizable, but music holds no pleasure. The literature suggests that trained musicians who get a CI are more likely to recover musical hearing to their satisfaction. Bilateral CIs sometimes improve hearing in noise and spatial localization of sound,^28^ so I am still considering a second CI. My gains through technology and training-induced learning, however, have enabled me to resume small group clinical neurology teaching around patient care and, most important, to delight in what my grandkids tell me.

## Conclusions

Progressive moderate to severe hearing loss challenges daily communication. The decline ensconces the listener in a web of verbal distortion, prunes the branches of personal connectivity with family, friends, and co-workers, and undercuts usual societal roles. Hearing aids and cochlear implants make functional communication more feasible for the majority of users, but neither amplification or electrical hearing are equivalent to normal cochlear acoustical hearing. The devices could become yet more acceptable and perhaps more effective if recipients routinely receive formal auditory/cognitive rehabilitation training from experts. Based on my experience, a routine clinical test of the maximum interpretable talking speed as a real-world metric of success for daily functioning with a CI should be valuable, just as walking speed over 10 m reflects the capacity for real-world mobility. Persons with moderate-severe hearing impairment can look forward to innovations that may bring yet more clarity to the colorations in our soundscapes.
